# Traumatic asphyxia due to blunt chest trauma: a case report and literature review

**DOI:** 10.1186/1752-1947-6-257

**Published:** 2012-08-30

**Authors:** Eleni Sertaridou, Vasilios Papaioannou, Georgios Kouliatsis, Vasiliki Theodorou, Ioannis Pneumatikos

**Affiliations:** 1 ICU department, University Hospital of Alexandroupolis, Dragana, Alexandroupolis 68100, Greece

## Abstract

**Introduction:**

Crush asphyxia is different from positional asphyxia, as respiratory compromise in the latter is caused by splinting of the chest and/or diaphragm, thus preventing normal chest expansion. There are only a few cases or small case series of crush asphyxia in the literature, reporting usually poor outcomes.

**Case presentation:**

We present the case of a 44-year-old Caucasian man who developed traumatic asphyxia with severe thoracic injury and mild brain edema after being crushed under heavy auto vehicle mechanical parts. He remained unconscious for an unknown time. The treatment included oropharyngeal intubation and mechanical ventilation, bilateral chest tube thoracostomies, treatment of brain edema and other supportive measures. Our patient’s outcome was good. Traumatic asphyxia is generally under-reported and most authors apply supportive measures, while the final outcome seems to be dependent on the length of time of the chest compression and on the associated injuries.

**Conclusion:**

Treatment for traumatic asphyxia is mainly supportive with special attention to the re-establishment of adequate oxygenation and perfusion; treatment of the concomitant injuries might also affect the final outcome.

## Introduction

Asphyxia is defined as any condition that leads to tissue oxygen deprivation [1]. Traumatic asphyxia is a type of mechanical asphyxia, where respiration is prevented by external pressure on the body, at the same time inhibiting respiratory movements and compromising venous return from the head. Conditions like compression of the chest and/or abdomen under a heavy weight and wedging of the body within a narrow space or large crowds have been reported [2]. A Valsalva maneuver is necessary before thoracic compression for development of the syndrome [3]. Usual autopsy findings include intense purple facial congestion and swelling with hemorrhagic petechiae of the face, the neck and upper chest, craniocervical cyanosis and subconjunctival hemorrhage.

## Case presentation

A 44-year-old Caucasian man was working under a car when the vehicle’s transmission system fell on his chest, squeezing his torso between the heavy item and the ground. After an unknown time, he was found in an unconscious state by a relative, who called for medical aid. It was estimated that at least one hour elapsed before our patient received medical care.

On arrival to our emergency department, our patient had a gasping breath without foreign bodies in his oronasal cavities, palpable regular pulses with a rate of 130 beats per minute and an arterial pressure of 80/40mmHg. On pulse oxymetry he had a saturation of 80% on room air. His Glasgow Coma Scale score was 8 (absent eye opening, unintelligible voice responses and limp withdrawal to painful stimuli), his papillae were isochoric and light reflexes were bilaterally present. Because of his altered consciousness and impending respiratory failure, our patient was urgently intubated and put under controlled mechanical ventilation.

The rest of the physical examination revealed that his face, the front part of his neck and the upper part of his chest were congested, edematous and covered with numerous petechiae, especially on the conjunctivae and the periorbital skin. In a later bedside ophthalmologic examination, mild bilateral periorbital swelling, severe bilateral subconjunctival hemorrhages, chemosis, mild exophthalmos and mild optic disc edema were observed. Ecchymotic bruises were also noted on the back part of his neck and the upper part of both shoulders. His tympanic membranes were clear and there were no mucosal hemorrhages of his upper airways.

**Figure 1:**
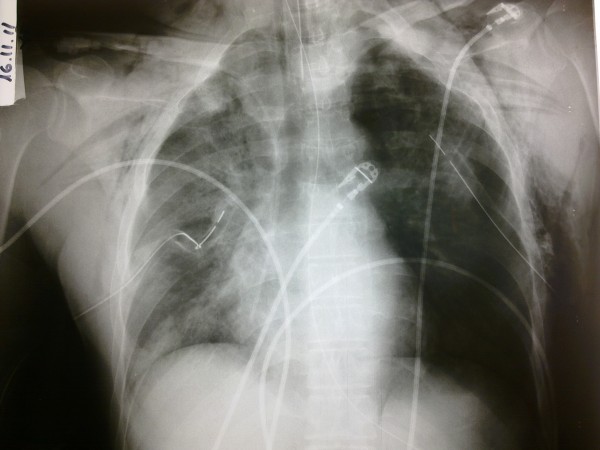
**Chest X-ray taken after tube thoracostomies were inserted.** Note multiple rib fractures, subcutaneous emphysema, multiple lung opacities, particularly on the right, corresponding to sites of lung contusion and residual pneumothorax on the left side.

Absence of breathing sounds over both lung apices in combination with palpable subcutaneous emphysema over his neck pointed towards the existence of bilateral pneumothorax. Moreover, bloody fluid was drained through the endotracheal tube, indicating possible lung contusions. The physical examination of his heart and abdomen was unremarkable and electrocardiogram was normal. Thoracic X-ray examination revealed bilateral pneumothorax and multiple rib fractures (Figure 1). In this respect, bilateral tube thoracostomies were inserted, draining air and blood and eliciting major improvement in his hemodynamic parameters. In subsequent X-rays, bilateral lung opacities were evident, which were consistent with the clinical suspicion of lung contusions. Fiberoptic bronchoscopy was not performed due to the bilateral pneumothorax. Subsequently, our patient was transferred to our intensive care unit (ICU). Arterial blood gases on admission to our ICU were: pH 7.246; partial pressure of carbon dioxide: 58.3mmHg; partial pressure of oxygen: 441mmHg; bicarbonate: 21.9mEq/L; oxygen saturation: 99.9%; and lactate: 1.1mmol/L while our patient was ventilated with a frequency of 15 breaths/min; tidal volume: 700mL; positive end-expiratory pressure: 5cmH_2_O; and fraction of inspired oxygen: 100%. His Acute Physiology and Chronic Health Evaluation II score was 14, while his past medical history was noted to be non-significant.

**Figure 2:**
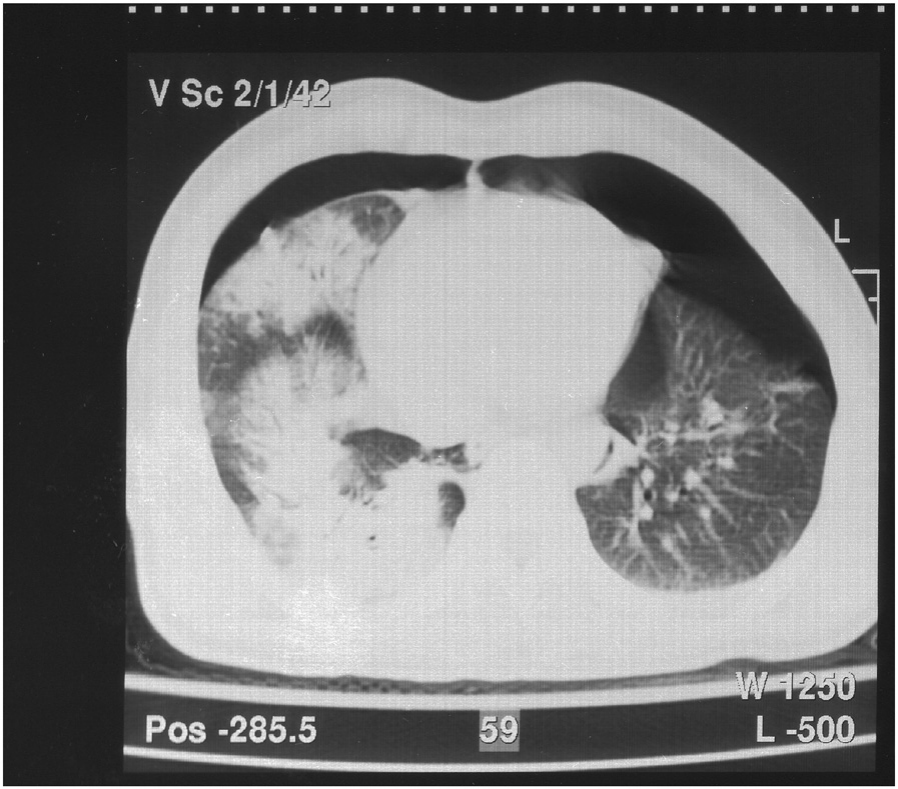
Computed tomography scan of the chest showing bilateral hemopneumothorax and multiple lung contusions, especially on the right.

Further work-up included radiological evaluation of his spine and limbs, which was unremarkable, a normal echocardiography, and head, neck, chest and abdomen computed tomography (CT). On the CT scan, a mild brain edema without signs of hemorrhage was observed, while CT of his chest revealed bilateral hemopneumothorax and sizeable bilateral lung contusions, particularly on his right lung (Figure 2).

Serum biochemistry included elevated levels (ten times above the upper limits of normal) of creatine phosphokinase, lactic dehydrogenase, aspartate aminotransferase and alanine aminotransferase. A urine analysis was normal (Table 1).

**Table 1:** Laboratory parameters of the patient during his stay in the intensive care unit

**Day**	**1**	**2**	**3**	**4**	**5**	**7**	**8**
Hematocrit (%)	45.5	-	-	31	31.2	31	33.3
Hemoglobin (g/dL)	15.5	-	-	10.5	10.7	10.9	11.5
Leukocytes (K/μL)	9390	-	-	9310	11,750	18,530	14,960
Platelets (K/μL)	245	-	-	189	212	244	303
International normalized ratio	1.07	-	-	-	-	-	1.25
Blood urea nitrogen (mg/dL)	34	27	22	38	38	33	35
Creatinine (mg/dL)	0.7	0.9	0.8	0.9	0.9	0.7	0.7
Aspartate aminotransferase (U/L)	**367**	**208**	**91**	**55**	**44**	**54**	30
Alanine aminotransferase (U/L)	**507**	**357**	**228**	**155**	**112**	**95**	66
Lactate dehydrogenase (U/L)	**879**	**545**	232	215	247	**447**	317
Creatine phosphokinase (U/L)	**924**	**1287**	**930**	**729**	**462**	**1391**	425
Creatine phosphokinase -MB (U/L)	**65**	**49**	17	16	15	**29**	13
γ-glutamyl transpeptidase (U/L)	22	19	18	26	**67**	**84**	72

Bold figures denote abnormal values.

In the ICU, our patient was ventilated with volume-control mode, with a tidal volume of 7mL/kg, frequency 10 to 12 per minute, positive end-expiratory pressure not exceeding 5cmH_2_O and a gas mixture that was quickly tapered to a fraction of inspired oxygen of 40%. The unimpeded ventilation, the swift restoration of hypercapnia, hypoxia and hemodynamics, the spontaneous containment of the tracheobronchial hemorrhage, the rapid radiographic improvement in the following days (disappearance of the opacities) and the quick recovery, simply by placing thoracostomies tubes, made the event of a potential rupture of major bronchial or arterial branch less plausible. Therefore, we did not proceed to further invasive diagnostic procedures, such as bronchoscopy, which would have added no more information towards the appropriate management of our patient and would even pose some risks.

Fluid resuscitation with crystalloids was copious, in order to prevent renal complications of a potential traumatic rhabdomyolysis. Special care for the brain edema was taken with mannitol administration and frequent neurologic assessments. Regarding his respiratory function, our patient improved swiftly, resulting in an uneventful extubation on the second day of ICU hospitalization. However, his neurologic status lagged behind, as he remained disoriented and agitated until the fourth day. Facial and thoracic petechiae gradually faded within the next three days. Serum aspartate aminotransferase, alanine aminotransferase, creatine phosphokinase and lactic dehydrogenase levels decreased to normal on the seventh day and our patient was discharged from the ICU and transferred to the thoracic surgical ward.

## Discussion

Crush asphyxia is caused by a sudden compressive trauma to the thoracoabdominal region and presents with facial cyanosis and edema, hyposphagmata and petechial hemorrhages of the face, neck and upper chest [4]. It is typically associated with transient ischemic neurological deficits and injuries to the thorax, abdomen and limbs.

Traumatic asphyxia was first described over 170 years ago, by Ollivier in his observations on the cadavers of people trampled upon during crowd upheavals in Paris on Bastille day [1]. Later, Perthes added some other characteristics, such as mental dullness, hyperpyrexia, hemoptysis, tachypnea and ‘contusion pneumonia’ to the initial description [1]. Other terms for this condition are Ollivier’s syndrome, Perthes’ symptom complex, compression cyanosis, traumatic cyanosis, cervicofacial static cyanosis and cervicofacial cutaneous asphyxia.

A review of the literature indicates that traumatic asphyxia is a rare condition, since it might go unrecognized or not be even reported. Laird and Borman found only seven cases out of 107,000 hospital and clinic patients in a 30-month period, of whom 75,000 had been involved in major accidents [5]. Dwek reported only one case out of a total of 18,500 accident victims in an area with heavy military traffic [6].

Our patient suffered from traumatic asphyxia due to prolonged compression between the ground and a sizeable heavy object, a mechanism quite common in similar published reports. In particular, cases of crush asphyxia are mainly a consequence of motor vehicle crashes, crushing among other bodies in a panicked crowd, entrapment beneath vehicles or falling down in a narrow space [7]. Other causes include injuries from machines and furniture, blast injury, a python tightened around the thorax and, rarely, deep-sea diving, weightlifting, epileptic seizures, difficult obstetric delivery and asthmatic attack. The typical range of the duration of compression is between two and five minutes [8]. The duration and the amount of pressure affect the outcome after traumatic asphyxia. Significant weight can be tolerated for a short time, whereas a relatively modest weight applied for a longer period may result in death [8]. In our case, the duration of compression could not be confirmed, but it is estimated as fairly long, although this is loosely consistent with the rapid and full recovery of the patient.

The diagnosis is reached from the physical appearance, clinical examination, history and trauma mechanism [3]. Superior vena cava (SVC) obstruction and basilar skull fracture have features that closely resemble the appearance of traumatic asphyxia. Yet, the history of traumatic injury should rule out SVC obstruction, while skull fractures are rare in traumatic asphyxia, unless the force of compression is applied to the head [7]. Our patient had no head injury, as verified by the imaging studies.

The exact pathophysiologic mechanism of traumatic asphyxia remains controversial. It is generally considered that a compressive force to the thoracoabdominal region together with the ‘fear response’ (deep breath and closing of the glottis) cause a huge increase in the central venous pressure. This induces reversal of venous blood flow from the heart through the SVC into the innominate and jugular veins of the head and neck. The back transmission of the elevated central venous pressure to the head and neck venules and capillaries, while arterial flow is continued, results into capillary stasis and rupture, producing the characteristic upper body petechial and subconjunctival hemorrhages [1]. These features are often more prominent on the eyelids, nose and lips [4]. The lack of petechiae in the lower body may be due to the compressive obstruction of the inferior vena cava in the chest or abdomen. Furthermore, the fact that the lower part of the body is protected from back transmission of venous pressure by a series of valves could be another mechanism, since the SVC, innominate and jugular veins have no valves [4].

Associated injuries, such as pulmonary, cardiac, neurologic, ophthalmic, abdominal and orthopedic trauma, were not apparent in our patient. As has been concluded from Rosato *et al*., cardiac injuries during traumatic asphyxia are extremely rare. Only two cases of cardiac contusion and one of ventricular rupture have been reported so far, within the last three years [9]. A normal electrocardiogram does not rule out blunt cardiac injury. Another rare consequence of traumatic asphyxia is delayed myocardial infarction due to coronary artery contusion [1]. Myoglobinuria, rhabdomyolysis and acute renal tubular necrosis (crush syndrome) present only in cases of associated injury and ischemia of large muscle groups [3].

After awakening and despite the normal findings on brain imaging, our patient was in a state of agitation and confusion that lasted for four days. According to Perthes, neurological injury in traumatic asphyxia includes cerebral hypoxia or anoxia, ischemia, venous hypertension, cerebral vascular congestion, rupture of small vessels, petechial hemorrhages and hydrostatic edema [2]. However, the rapid full recovery discouraged us from requesting further brain imaging studies, such as magnetic resonance imaging, that were not expected to influence the treatment plan. The vision may be affected with the same mechanism: retinal hemorrhage, retrobulbar hemorrhage and vitreous exudates (Purtscher’s retinopathy) [10]. A hearing deficit can be caused by edema of the Eustachian tubes, or a hemotympanum. Other neurologic manifestations of the syndrome are loss of consciousness, prolonged but self-limiting confusion, disorientation, agitation, restlessness, seizures, visual disturbances, blurred vision, papillary changes, optic nerve atrophy, exophthalmos, diplopia and hearing loss [10]. Often, the neurologic status improves during transfer to the emergency room [8]. The suggested mechanism for loss of consciousness and prolonged confusion associated with traumatic asphyxia includes cerebral hypoxia, ischemia and venous hypertension, which lead to cortical dysfunction. This dysfunction resolves within the following 24 to 48 hours. Intracranial hemorrhage has seldom ever been evident in a patient [8]. CT scans of the brain are usually normal, whereas in fatal cases, autopsy shows only petechiae and congestion, suggesting brain injury at the cellular level [1].

Despite the dramatic appearance of the ‘ecchymotic mask’, mortality in crush asphyxia is low. However, it may be influenced by the severity, nature and duration of the compressive force and the presence of concomitant injuries, which can be useful markers of the severity of compression [2]. The proposed algorithm for the management of all trauma patients on arrival and during the initial phases of treatment is the ABCDE (Airway, Breath, Circulation, Disability, Environment) algorithm, described in the Advanced Trauma Life Support guidelines by the American College of Surgeons Committee on Trauma. The outcome is improved by airway control and cervical spine protection, rapid restoration of ventilation, oxygenation and circulation by thoracic decompression, fluid resuscitation and prevention of renal complications secondary to rhabdomyolysis and other secondary causes [1]. Management of these patients may be complicated by severe upper airway edema, and the possibility of a difficult intubation should thus be considered early. The prognosis is good if the patient survives the initial few hours following injury, although a prolonged thoracic compression could lead to cerebral anoxia and permanent neurological sequelae [3].

## Conclusion

Optimal management of traumatic asphyxia must focus on early recognition of this entity based upon the classic physical signs and the mechanism of injury. Resuscitation efforts should include rapid administration of oxygen with effective ventilation and fluid resuscitation, and must focus on reversing hypoxia and prevent further tissue damage.

## Consent

Written informed consent was obtained from the patient for publication of this case report and accompanying images. A copy of the written consent is available for review by the Editor-in-Chief of this journal.

## Competing interests

The authors declare that they have no competing interests.

## Authors’ contributions

ES, GK and VT managed the patient, reviewed the literature and contributed to the preparation of the manuscript; VP and IP reviewed the manuscript and contributed to its final form. All authors read and approved the final manuscript.

## Authors’ information

Eleni Sertaridou, MD is a Surgeon-Intensivist at the ICU University Hospital of Alexandroupolis, Alexandroupolis, Greece. Vasilios Papaioannou, MD, MSc, PhD is an Assistant Professor of Critical Care Medicine at the ICU University Hospital of Alexandroupolis, Alexandroupolis, Greece. Georgios Kouliatsis, MD is a Pulmonologist-Intensivist at the ICU University Hospital of Alexandroupolis, Alexandroupolis, Greece. Vasiliki Theodorou, MD is an Anaethesiologist-Intensivist at the ICU University Hospital of Alexandroupolis, Alexandroupolis, Greece. Ioannis Pneumatikos, MD, PhD, FCCP is a Professor of Critical Care Medicine at the Democritus University of Thrace and Head of ICU, University Hospital of Alexandroupolis, Alexandroupolis, Greece.
